# MRI-Based Risk Assessment for Incomplete Resection of Brain Metastases

**DOI:** 10.3389/fonc.2022.873175

**Published:** 2022-05-16

**Authors:** Tizian Rosenstock, Paul Pöser, David Wasilewski, Hans-Christian Bauknecht, Ulrike Grittner, Thomas Picht, Martin Misch, Julia Sophie Onken, Peter Vajkoczy

**Affiliations:** ^1^ Department of Neurosurgery, Charité – Universitätsmedizin Berlin, Corporate Member of Freie Universität Berlin, Humboldt-Universität zu Berlin, and Berlin Institute of Health, Berlin, Germany; ^2^ Berlin Institute of Health at Charité – Universitätsmedizin Berlin, BIH Biomedical Innovation Academy, BIH Charité Digital Clinician Scientist Program, Berlin, Germany; ^3^ Institute of Neuroradiology, Charité – Universitätsmedizin Berlin, Corporate Member of Freie Universität Berlin, Humboldt-Universität zu Berlin, and Berlin Institute of Health, Berlin, Germany; ^4^ Institute of Biometry and Clinical Epidemiology, Charité – Universitätsmedizin Berlin, Corporate Member of Freie Universität Berlin, Humboldt-Universität zu Berlin, and Berlin Institute of Health, Berlin, Germany; ^5^ Cluster of Excellence: “Matters of Activity. Image Space Material,” Humboldt University, Berlin, Germany

**Keywords:** brain metastasis, magnetic resonance imaging (MRI), neurosurgical resection, extent of resection, (GTR) gross total resection, (STR) subtotal resection

## Abstract

**Object:**

Recent studies demonstrated that gross total resection of brain metastases cannot always be achieved. Subtotal resection (STR) can result in an early recurrence and might affect patient survival. We initiated a prospective observational study to establish a MRI-based risk assessment for incomplete resection of brain metastases.

**Methods:**

All patients in whom ≥1 brain metastasis was resected were prospectively included in this study (DRKS ID: DRKS00021224; Nov 2020 – Nov 2021). An interdisciplinary board of neurosurgeons and neuroradiologists evaluated the pre- and postoperative MRI (≤48h after surgery) for residual tumor. Extensive neuroradiological analyses were performed to identify risk factors for an unintended STR which were integrated into a regression tree analysis to determine the patients’ individual risk for a STR.

**Results:**

We included 150 patients (74 female; mean age: 61 years), in whom 165 brain metastases were resected. A STR was detected in 32 cases (19.4%) (median residual tumor volume: 1.36ml, median EOR_rel_: 93.6%), of which 6 (3.6%) were intended STR (median residual tumor volume: 3.27ml, median EOR_rel_: 67.3%) - mainly due to motor-eloquent location - and 26 (15.8%) were unintended STR (uSTR) (median residual tumor volume: 0.64ml, median EOR_rel_: 94.7%). The following risk factors for an uSTR could be identified: subcortical metastasis ≥5mm distant from cortex, diffuse contrast agent enhancement, proximity to the ventricles, contact to falx/tentorium and non-transcortical approaches. Regression tree analysis revealed that the individual risk for an uSTR was mainly associated to the distance from the cortex (distance ≥5mm vs. <5mm: OR 8.0; 95%CI: 2.7 – 24.4) and the contrast agent patterns (diffuse vs. non-diffuse in those with distance ≥5mm: OR: 4.2; 95%CI: 1.3 – 13.7). The preoperative tumor volume was not substantially associated with the extent of resection.

**Conclusions:**

Subcortical metastases ≥5mm distant from cortex with diffuse contrast agent enhancement showed the highest incidence of uSTR. The proposed MRI-based assessment allows estimation of the individual risk for uSTR and can help indicating intraoperative imaging.

## Introduction

Metastasis to the brain is a common complication of systemic cancer with an incidence of 20-40%, with lung cancer (20–56% of patients), breast cancer (5–20%) and melanoma (7–16%) representing the most common primary tumor entities (1–3). They can severely compromise patients’ quality of life due to symptoms (such as focal neurologic deficits and epileptic seizures) and can directly lead to death in 31% - 52% of these patients (2, 4, 5).

Due to novel targeted therapies, systemic control is more often achieved, resulting in prolonged survival and thus increasing the incidence of cerebral metastases (4, 6). Since the blood brain barrier and the specific tumor microenvironment limit the efficacy of many therapeutic agents, surgical resection and radiation therapy are the primary options for local control (3, 7–9). Local recurrence could be related to subtotal resection, emphasizing the importance of objectively assessing the extent of resection (EOR). So far, EOR in brain metastases has only been analyzed in retrospective studies. They showed that the rate of gross total resection (GTR) is lower than assumed in the past, and that surgical assessment alone cannot serve as an objective measure (10–13).

The use of intraoperative imaging such as MRI (iopMRI) or ultrasound (iopUS) is well established in primary brain tumors to improve the extent of resection (14–16). The cost of an iopMRI unit ranges from $3 million to $7 million, plus the cost of remodeling the operating room. Since radiological staff is also required, iopMRI is not available in every hospital (17). In contrast, iopUS is a relatively cost-effective technology, but one that requires significant expertise and thus also hinder its application (14). The estimated incremental cost per case are $1813 for iopMRI and $333 for iopUS (18). Rational resource allocation and individual evaluation (considering the advantages and disadvantages of iopMRI and iopUS) are necessary to accurately identify cases that may benefit.

The aim of this prospective study was to identify risk factors for a subtotal brain metastasis resection. In addition, a statistical model based on patient characteristics and the preoperative MRI will be established to determine the patients’ individual risk for a subtotal resection (STR).

## Methods

### Study Design

This prospective, observational study started in November 2020, was approved by the Ethics Committee of the Charite - Universitätsmedizin Berlin (EA2/232/20) and is in accordance with the STROBE Guidelines (19) and the ethical standards of the Declaration of Helsinki. This unicentric analysis is part of a prospective brain metastasis database, registered with the German Clinical Trials Register (DRKS-ID: DRKS00021224).

### Patients

A prospective database of all patients (age ≥18 years) who underwent neurosurgical resection of one or more brain metastases between 11/2020 and 11/2021 was established using the Research Electronic Data Capture (REDCap) platform (20). Exclusion criteria were the missing of a (preoperative) MR imaging (e.g. due to an emergency craniotomy or a non-MRI capable pacemaker). All patients were examined preoperatively and postoperatively for the presence of neurologic deficits by the treating neurosurgical team. The degree of disability/dependence in daily living was estimated using the Karnofsky Performance Scale (KPS) and the modified Rankin Scale (mRS). The primary tumor and the presence or number of intra/extracranial metastases were recorded in addition to oncological history.

### Neurosurgical Resection

All operations were performed in microsurgical technique using the microscope and neuronavigation. The treating neurosurgeon decided about using intraoperative neuromonitoring (IOM) to ensure the integrity of the motor system based on a previously published risk stratification (21, 22). Warning criteria were a decrease in MEP amplitudes >50% and a subcortical stimulation intensity <5mA which we described in detail elsewhere (23). No awake craniotomies were performed in this series. If a metastasis was intentionally resected incompletely (e.g., because of infiltration of the skull base or because of eloquent location such as near the motor cortex), this was documented separately. If residual tumor was found postoperatively, it was at the discretion of the treating neurosurgeon to decide on re-resection based on the size of the residual tumor and the presence of further metastases.

### MR Imaging

**Figure 1:**
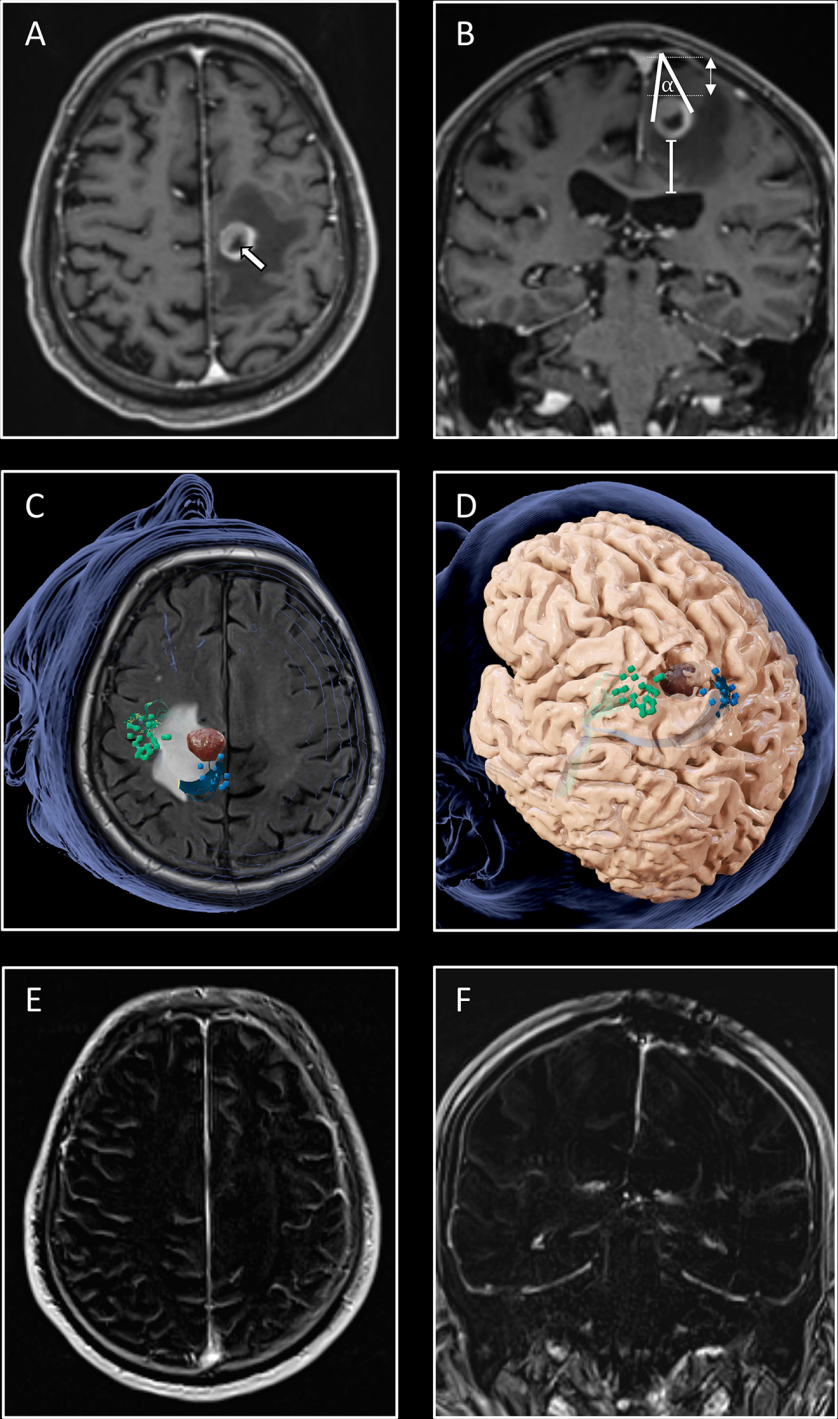
Illustration of the workflow. An 80-year-old patient presented to the emergency department with inability to walk and with leg accentuated hemiparesis. MRI **(A, B)** revealed a circumscribed contrast agent enhancing tumor with a central necrosis (white arrow) in the left hemisphere in precentral locaton. Volume of the tumor (2.5ml), edema (26ml), the distance from the cortex (white double arrow; 19mm) and from the ventricle (white line; 29mm) were measured. The tumor-cortex angle (α; 35°) was determined only for subcortical metastases, with the angle legs defined by the largest diameter and the apex defined by the cortical approach. The cortical nTMS motor mapping **(C)** for the upper (green bullets) and lower extremity (blue bullets) showed approximation of the tumor to the motor cortex. The minimum distance between the tumor and the corticospinal tract was 8mm **(D)**, indicating a motor-associated location with higher risk for postoperative motor worsening (22). An interhemispheric approach under intraoperative neuromonitoring guidance was chosen for tumor removal. The patient recovered postoperatively with ability to walk again. No residual contrast agent enhancement was detected in the postoperative T1 subtraction sequences, confirming a GTR **(E, F)**.

Following the EANO guidelines (24), all included patients received a 1.5T or 3T MRI scan before and after surgery (≤48h). T1-weighted 3D gradient echo sequences (MP-Rage, 1mm isotropic voxel size) with gadoterate meglumine (0.2ml/kg body weight; Dotarem; Guerbet; France) were used to characterize the metastases. In addition, T2 sequences were used to assess perifocal edema, SWI sequences (or cerebral computer tomography if available) to analyze hemorrhage, and diffusion-weighted imaging to assess ischemia. The following characteristics were analyzed preoperatively with the planning software Elements (Brainlab AG): number of brain metastases, localization, volume, tumoral cyst/necrosis, perifocal edema, hemorrhage, opening of a ventricle probable (threshold ≤5 mm), contact with dura/distance to brain surface, tumor-cortex angle (**Figure 1**), signs of meningeosis and detection of hydrocephalus. The contrast agent patterns were also evaluated to determine whether a metastasis was circumscribed if it showed a) clear margin to the healthy brain tissue and b) round, circumscribed contrast medium uptake without filiform/finger-shaped spreading. If the criteria were not met or there was an unclear assessment, the contrast agent patterns were classified as diffuse.

Patients with presumed motor- or language-associated tumor location underwent navigated transcranial magnetic stimulation (nTMS) mapping for localization of cortical functional areas (22, 25). These functional data were used as seeding regions for the diffusion tensor imaging (DTI) tractography to visualize the corticospinal tract (for motor cases) and/or the language network as previously published elsewhere (22, 25). The institutional’s neuroimaging experts and the treating neurosurgical team decided about a motor-/language-associated tumor location based on the individual risk analysis (22, 26).

An independent neuroradiologist with more than 15 years of experience validated and reviewed the extent of resection (EOR) analysis, which was evaluated primarily by an interdisciplinary panel of neurosurgeons (including the treating neurosurgical team) and neuroradiologists. The T1 subtraction sequences (27) were used to divide the cases into gross total resection (GTR = complete resection of the contrast-enhancing tumor parts without hints of residual tumor tissue), intended subtotal resection (iSTR = intentionally left tumor parts, e.g. due to proximity to the corticospinal tract) and unintended subtotal resection (uSTR). Any contrast uptake on the postoperative MRI was considered as residual tumor. In these cases, the volume as well as the relative extent of resection [EOR_rel_ = 1 – (volume_tumor postop._/volume_tumor preop._)] were measured.

### Statistical Analysis

SPSS Statistics 25.0 (IBM Corp., Armonk, N.Y., USA) and R (R Core Team) were used to analyze the data in collaboration with the Institute of Biometry and Clinical Epidemiology – Charité. For the bivariate analysis, we used descriptive statistics and standardized mean differences (SMD) based on approaches of Austin (28) and Yang & Dalton (29) for dichotomous and multinomial variables to examine extent of group differences between those patients with GTR compared to those with uSTR. In this exploratory study, the interpretation of the results is based on the effect size estimates. To account for the dependency of measures in the same individuals, the calculation of the significance level α was performed cautiously, as group differences were tested with binary logistic generalized estimating equations (GEE). For the multiple analysis (aiming to determine relevant risk factors for an uSTR), we used the package rpart of the R statistical platform (4.0.2) to calculate regression trees with 10-fold cross validation (minimum number of patients in node for split: 18, minimum number of patients in bucket: 6, splitting index: Gini coefficient). The following independent factors were tested as potential risk factors.: side/location of brain metastases, tumor volume, distance from cortex, metastasis recurrency, presence of cystic/necrotic parts, bleeding, peritumoral edema, hydrocephalus, proximity to the ventricular system (cut-off value: 5mm), contract to falx/tentorium, signs for leptomeningeal disease, contrast agent patterns, tumor-cortex angle and the used surgical approach.

## Results

### Patients Sample

A total of 150 patients were included, in whom 165 metastases were resected. A detailed description of the patient characteristics is outlined in **Table 1**. The study population had a mean age of 61 years (range: 28-86 years; IQR: 14 years), with equal proportions of women and men (74 women; 49.3%).

**Table 1:** Patient Sample.

**n**	150
**Age** in years as mean [SD], (range)	61 [12], (27-86)
**Sex**	
** Female**	74 (49.3%)
** Male**	76 (50.7%)
**Preoperative clinical status**	
**Epilepsy**	19 (12.7%)
**Neurological deficit**	123 (82.0%)
Paresis	23 (15.3%)
Aphasia	10 (6.7%)
Cranial nerve deficit	10 (6.7%)
Vertigo and ataxia	25 (16.7%)
Reduced vigilance	13 (8.7%)
Sensory disturbance	8 (5.3%)
Multiple deficits	34 (22.7%)
**KPS**	
Good performance (90%-100%)	70 (46.7%)
Intermediate performance (70%-80%)	60 (40.0%)
Poor performance (< 70%)	20 (13.3%)
**mRS** as median [IQR], (range)	1 [1-2], (0-4)
**Tumor history**	
** Primarius**	
** **Lung	61 (40.7%)
Breast	27 (18%)
Melanoma	19 (12.7%)
Kidney	9 (6.0%)
Other	34 (22.7%)
** Initial diagnosis**	39 (26.0%)
** Diagnosis by routine staging**	16 (10.7%)
**Extracranial metastases**	70 (46.7%)
** History of preop. chemotherapy**	59 (39.3%)
** History of preop. immunotherapy**	58 (38.7%)
**Number of brain metastases per patient**	
Singular (1)	95 (63.3%)
Oligometastases (2-4)	40 (26.7%)
Multiple (>4)	15 (10.0%)
**Number of resected metastases**	
1	136 (90.67%)
2	13 (8.67%)
3	1 (0.67%)

Table 1 presents the patients characteristics. KPS, Karnfosky Performance Status Scale; mRS, Modified Rankin Scale.

One hundred and twenty-three patients (82.0%) had a preoperative neurologic deficit, with paresis (23 cases; 15.3%) and vertigo/ataxia (25 cases; 16.7%) being the most common entities. The preoperative neurologic deficits improved postoperatively in 53 of the 123 patients (43.1%). A new postoperative neurological deficit occurred in 14 cases (9.3%), of which 8 (5.3%) had new paresis, 3 (2.0%) had aphasia and 5 (3.3%) had other deficits such as ataxia. The new deficits were transient in 6 cases (4.0%) and permanent in 2 cases (1.3%), whereas no data could be obtained for 6 patients (4.0%) (loss of follow-up). Surgical revisions were required in 13 cases (8.7%) due to wound healing disorder (8 cases; 5.3%), postoperative hydrocephalus (2 cases; 1.3%) or postoperative bleeding (3 cases; 2.0%). Two patients (1.3%) developed a postoperative meningitis.

### Metastases Characteristics

Most metastases were located supratentorially (125; 75.8%) and here most frequently in the frontal lobe (41; 24.8%). The metastases had a median tumor volume of 9.0ml (range 0.3ml – 124.0ml; IQR: 13.35ml) and an overall equal incidence in the right and left (cerebellar) hemispheres (78 cases each (47.3%)). In 39 cases (26.0%), the brain metastasis was the initial manifestation of cancer, with lung carcinoma being the most common primarius. Fourteen cases (8.5%) were metastatic recurrence. Seventy-three metastases (44.2%) grew to the cortex surface, whereas 92 metastases (55.8%) were located solely subcortically.

### Extent of Resection Analysis

A gross total resection (GTR) was achieved in 133 metastases (80.6%). In cases of a STR (32 cases; 19.4%), the median residual tumor volume was 1.36ml (range 0.03ml – 8.70ml; IQR: 1.14ml) and the median EOR_rel_ was 93.6% (range 48% - 99%; IQR: 0.21). In 6 metastases (3.6%), an iSTR was performed for the following reasons: motor eloquent location (4 cases; 2.4%), skull base infiltration (1 case; 0.6%) and brainstem infiltration (1 case; 0.6%). The postoperative MRI revealed an uSTR in 26 metastases (15.8%), in which 2 metastases (1.2%; 7.7% of uSTR) were decided to undergo a re-resection and 24 metastases (14.6%; 92.3% of uSTR) were no re-resected. Residual tumor volume was significantly larger in the iSTR group (median absolute volume 3.27ml, range 1.45ml – 8.70ml; IQR: 4.01ml) compared to the uSTR group (median 0.64ml; range 0.03ml – 2.46ml; IQR: 0.89ml) (p <.001; SMD = 1.80) which could also be observed with the relative extent of resection (uSTR: median EOR_rel_ = 94.7%, range 52% - 99%, IQR: 0.17; iSTR: median EOR_rel_ = 67.3%, range 48% - 93%, IQR: 0.36; p = .003, SMD = 1.38).

**Figure 2:**
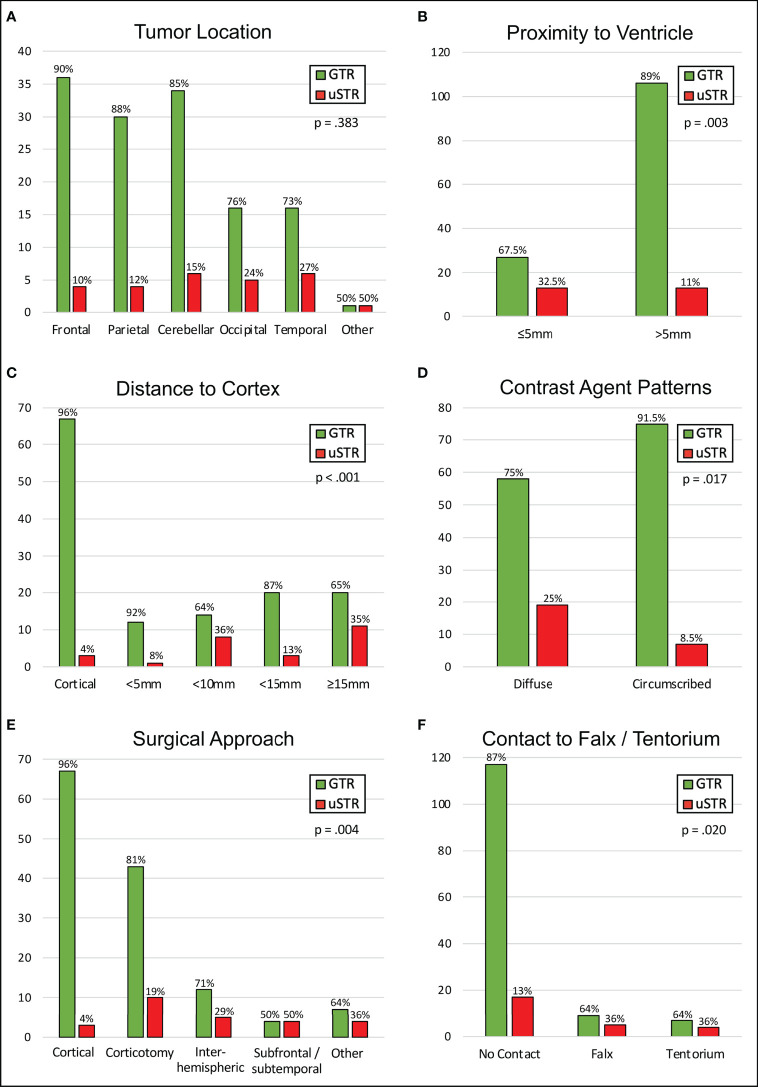
Univariate analysis of the EOR. Bar graphs showing the relationship between the EOR and the tumor location **(A)**, the distance to the ventricle system **(B)**/to the cortex **(C)**, the contrast agent patterns **(D)**, the chosen surgical approach **(E)** and to the falx-tentorium contact **(F)**. GTR, gross total resection, uSTR, unintended subtotal resection.

**Table 2** shows the bivariate analysis of the metastases’ characteristics and the EOR to identify risk factors for an uSTR. The iSTR were excluded from this analysis (**Supplement 1**). Cortical metastases had a low risk of an uSTR while subcortical locations ≥5mm distant from cortex and non-transcortical approaches were associated with higher risk of uSTR (**Figure 2**; distance from cortex: p <.001, SMD = 1.08; surgical approach: p = .004; SMD = 1.03). Patients in whom the metastasis showed well-defined/circumscribed contrast agent enhancement were less likely to have an uSTR (**Figure 2**, p = .017, SMD = 0.67). Falx/tentorium contact and proximity to the ventricles were also associated with a higher risk of uSTR (**Figure 2**, falx/tentorium contact: p = .020, SMD = 0.55; proximity to ventricles: p = .003, SMD = 0.65). Patients with an intratumoral cyst/necrosis were more likely to have an uSTR (**Table 2**, p = .063; SMD = 0.40). Neither other metastases’ characteristics (such as the preoperative tumor volume) nor the application of IOM showed any substantial association to the risk of uSTR (**Table 2**).

**Table 2:** Bivariate Analysis for Unintended Subtotal Resections.

n	total	Extent of resection		SMD	p
		GTR	uSTR		
	159	133	26		
**Side of resected metastases**				0.17	.657^A^
Right	77 (48.4%)	65 (48.9%)	12 (46.2%)		
Left	75 (47.2%)	63 (47.4%)	12 (46.2%)		
Midline	7 (4.4%)	5 (3.8%)	2 (7.7%)		
**Location**				0.50	.383^A^
Frontal	40 (25.2%)	36 (27.1%)	4 (15.4%)		
Parietal	34 (21.4%)	30 (22.6%)	4 (15.4%)		
Cerebellar	40 (25.2%)	34 (25.6%)	6 (23.1%)		
Occipital	21 (13.2%)	16 (12.0%)	5 (19.2%)		
Temporal	22 (13.8%)	16 (12.0%)	6 (23.1%)		
Other	2 (1.3%)	1 (0.8%)	1 (3.8%)		
**Volume**				0.11	.968^A^
≤5ml	41 (25.8%)	35 (26.3%)	6 (23.1%)		
≤10ml	47 (29.6%)	39 (29.3%)	8 (30.8%)		
≤15ml	21 (13.2%)	18 (13.5%)	3 (11.5%)		
>15ml	50 (31.4%)	41 (30.8%)	9 (34.6%)		
**Recurrent metastasis**				0.19	.456^A^
No	147 (92.5%)	122 (91.7%)	25 (96.2%)		
Yes	12 (7.5%)	11 (8.3%)	1 (3.8%)		
**Cystic/necrotic parts**				0.40	.063^A^
No	87 (54.2%)	77 (57.9%)	10 (38.5%)		
Yes	72 (45.3%)	56 (42.1%)	16 (61.5%)		
**Bleeding**				0.12	.582^A^
No	135 (84.9%)	112 (84.2%)	23 (88.5%)		
Yes	24 (15.1%)	21 (15.8%)	3 (11.5%)		
**Edema**				0.11	.604^A^
No	31 (19.5%)	25 (18.8%)	6 (23.1%)		
Yes	128 (80.5%)	108 (81.2%)	20 (76.9%)		
**Distance to ventricle**				0.65	**.003^A^**
≤5mm	40 (25.2%)	27 (20.3%)	13 (50.0%)		
>5mm	119 (74.8%)	106 (79.7%)	13 (50.0%)		
**Contact to falx/tentorium**				0.55	**.020^A^**
No	134 (84.3%)	117 (88.0%)	17 (65.4%)		
Falx	14 (8.8%)	9 (6.8%)	5 (19.2%)		
Tentorium	11 (6.9%)	7 (5.3%)	4 (15.4%)		
**LMD**				0.07	.758^A^
No	151 (95.0%)	126 (94.7%)	25 (96.2%)		
Yes	8 (5.0%)	7 (5.3%)	1 (3.8%)		
**Occlusive hydrocephalus**				0.24	.313^A^
No	145 (91.2%)	120 (90.2%)	25 (96.2%)		
Yes	14 (8.8%)	13 (9.8%)	1 (3.8%)		
**Contrast agent patterns**				0.67	**.017^A^**
Diffuse	77 (48.4%)	58 (43.6%)	19 (73.1%)		
Circumscribed	82 (51.6%)	75 (56.4%)	7 (26.9%)		
**Cortical vs. subcortical location**				0.93	**.001^A^**
Cortical	70 (44.0%)	67 (50.4%)	3 (11.5%)		
Subcortical	89 (56.0%)	66 (49.6%)	23 (88.5%)		
**Distance from cortex**				1.08	**<.001^A^**
0mm	70 (44.0%)	67 (50.4%)	3 (11.5%)		
<5mm distant cortex	13 (8.2%)	12 (9.0%)	1 (3.8%)		
<10mm distant cortex	22 (13.8%)	14 (10.5%)	8 (30.8%)		
<15mm distant cortex	23 (14.5%)	20 (15.0%)	3 (11.5%)		
≥15mm distant cortex	31 (19.5%)	20 (15.0%)	11 (42.3%)		
**Tumor-cortex angle**				0.19	.873^A^
0 - ≤37.5°	22 (24.7%)	17 (25.8%)	5 (21.7%)		
37.5% - ≤47°	24 (27.0%)	17 (25.8%)	7 (30.4%)		
47° - ≤62.5°	23 (25.8%)	18 (27.3%)	5 (21.7%)		
>62.5°	20 (22.5%)	14 (21.2%)	6 (26.1%)		
**Surgical Approach**				1.03	**.004^A^**
Cortical Metastasis	70 (44.0%)	67 (50.4%)	3 (11.5%)		
Corticotomy	53 (33.3%)	43 (32.3%)	10 (38.5%)		
Interhemispheric	18 (10.7%)	12 (9.0%)	5 (19.2%)		
Subfrontal/subtemporal	8 (5.0%)	4 (3.0%)	4 (15.4%)		
Other (e.g. retrosigmoidal)	11 (6.9%)	7 (5.3%)	4 (15.4%)		
**Motor Associated Location**				0.07	.739^A^
No	131 (82.4%)	109 (82.0%)	22 (84.6%)		
Yes	28 (17.6%)	24 (18.0%)	4 (15.4%)		
**Language Associated Location**				0.02	.942^A^
No	140 (88.1%)	117 (88.0%)	23 (88.5%)		
Yes	19 (11.9%)	16 (12.0%)	3 (11.5%)		
**IOM application**				0.18	.372^A^
** No**	124 (78.0%)	102 (76.7%)	22 (84.6%)		
** Yes**	35 (22.0%)	31 (23.3%)	4 (15.4%)		

Table 2 analyzes the relationship of metastases’ characteristics to the extent of resection. For this analysis, intended subtotal resections (n = 6) were excluded to avoid biasing the risk assessment for unplanned resections. The tumor-cortex angles were only calculated for subcortical metastases (n = 95) and grouped according to percentiles distribution. ^A^, binary logistic generalized estimating equation (GEE). GTR, gross total resection; uSTR, unplanned subtotal resection; SMD, standardized mean difference; LMD, leptomeningeal disease; IOM, intraoperative neurophysiological monitoring. P values printed in bold indicate statistical significance.

### Regression Tree Analysis

**Figure 3:**
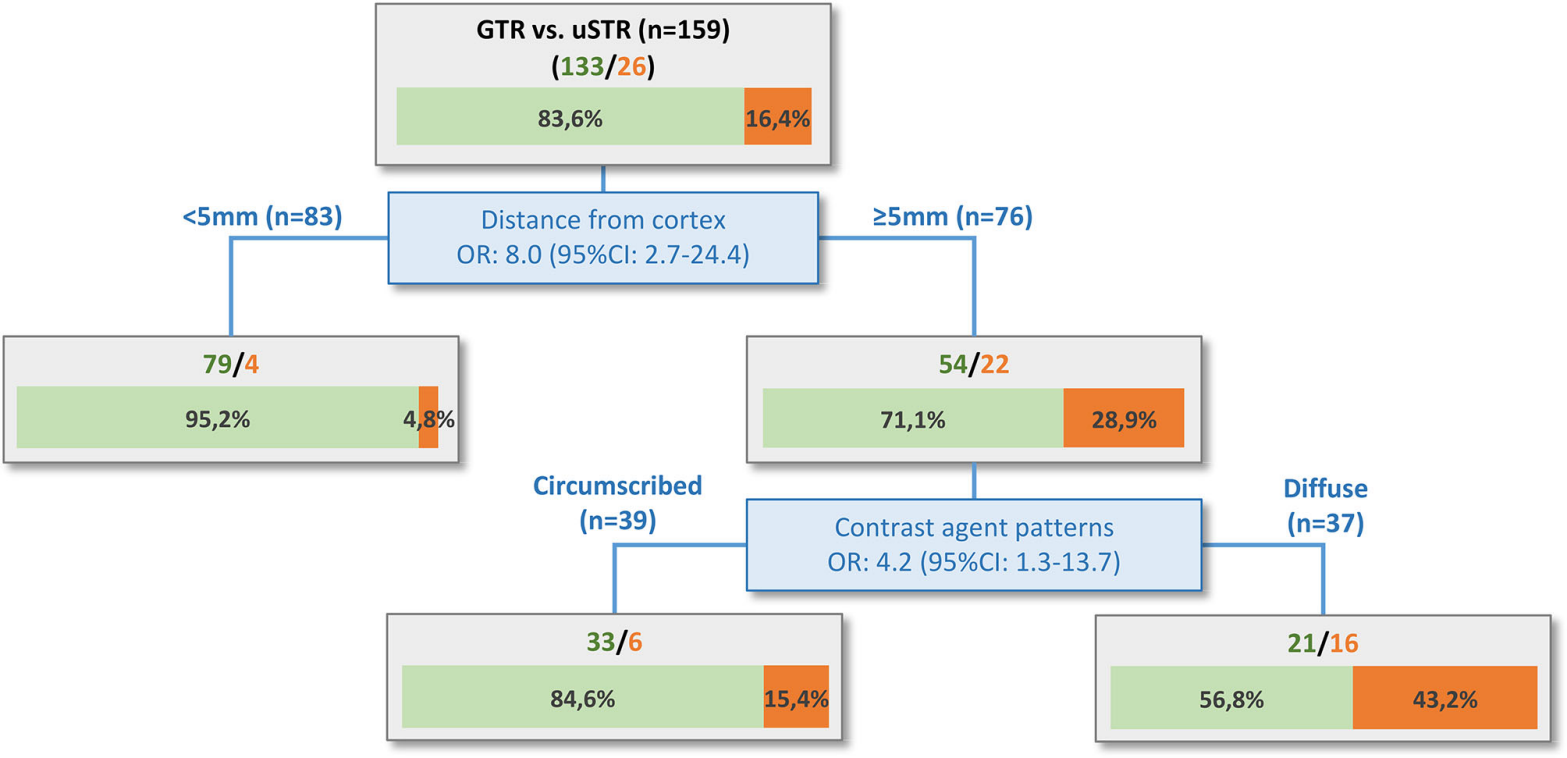
Regression tree analysis. The distance of the metastasis from the cortex is the most relevant risk factor, with 5mm being the cut-off value for higher (28.9%) or lower (4.8%) probability of an uSTR. The risk for patients with metastasis ≥5mm distant from cortex can be further stratified based on the contrast agent patterns. Subcortical metastases with clear margins to the healthy brain tissue and round, circumscribed contrast agent enhancement without filiform/finger-shaped spreading had a lower risk (15.4%) for an uSTR. In contrast, subcortical metastases with diffuse contrast agent enhancement had the highest risk (43.2%) for an uSTR. The tree diagram also shows the individual odds ratios (OR) with 95% confidence interval (95%CI) of the parameters.

**Figure 4:**
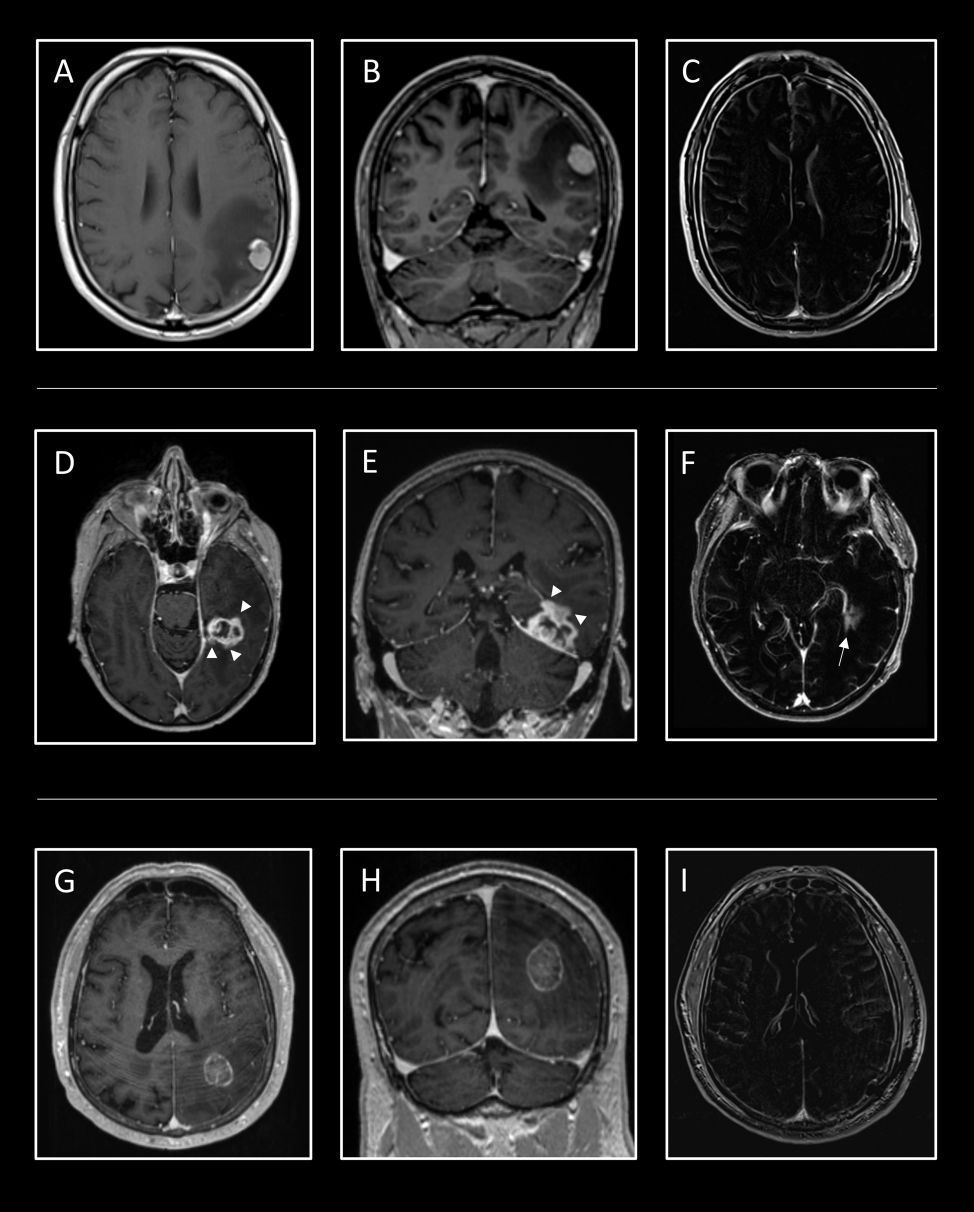
Case examples for the application of the regression tree. In the first line, the case of a 56-year-old man is shown who presented to the emergency department with an epileptic seizure and aphasia. Cerebral MRI showed a homogeneous contrast-enhancing cortical tumor in the parietal lobe with a volume of 2.2ml and extensive perifocal edema **(A, B)**. The risk of uSTR is very low at 4.8%, thus a GTR was confirmed in the postoperative T1 subtraction sequence **(C)**. Additionally, the preoperative aphasia improved. The second case shows a 69-year-old woman whose temporal metastasis of esophageal carcinoma grew progressively to 9.5ml despite radiation **(D, E)**. Neurological examination revealed mild aphasia. Detailed analysis revealed an irregular/diffuse contrast agent enhancement (white arrowheads), which, in combination with a distance of 15.4mm from the cortex, indicates a high risk of an uSTR at 43.2%. The aphasia improved postoperatively, but a residual tumor (white arrow) of 0.6ml (EOR_rel_ = 94%) was detected **(F)**. At the bottom, the MRI of a 70-year-old man who suffered from ataxia and visual disturbance presents a subcortical tumor in the parietal lobe with a volume of 9.0ml **(G, H)**. The tumor has circumscribed contrast agent enhancement, with 11mm distant from the cortex, thus the risk for uSTR is moderate at 15.4%. Postoperatively, the ataxia and visual impairment improved and a GTR was obtained **(I)**.

The before mentioned parameters were included in the regression tree analysis (**Figure 3**). The distinction between cortical and subcortical location (with a calculated threshold of 5mm) was the most impacting risk parameter (rate of uSTR: 4.8% vs. 28.9%). The first node of the tree (distance from cortex ≥ 5mm) shows a sensitivity of 84.6%, specificity of 59.4%, positive predictive value (PPV) of 28.9% and a negative predictive value (NPV) of 95.2%. In case of a subcortical metastasis (≥ 5mm distant from cortex), the regressions tree analysis revealed that a diffuse contrast agent enhancement was associated with a risk for an uSTR (43.2% vs. 15.4%). The analysis of the entire regression tree with both nodes shows an accuracy of 80.5%, a sensitivity of 61.5%, a specificity of 84.2%, a PPV of 43.2% and a NPV of 91.8%. The clinical applicability of the regression tree is illustrated in **Figure 4**.

## Discussion

### Main Finding of the Study

This is the first study which prospectively analyzed the extent of resection in brain metastases. A GTR was achieved in 80.6%, whereas an iSTR was observed in 3.6% and an uSTR in 15.8%. The interdisciplinary neurosurgical and neuroradiological analysis of the preoperative MRIs allowed us to identify risk factors for uSTR, with patients having a subcortical metastasis (≥5mm distant from cortex) with diffuse contrast enhancement being at highest risk. Regression tree analysis allows easy clinical application to decide whether to use intraoperative imaging techniques such as iopMRI or iopUS to allow complete resection.

Preoperative neurologic deficits improved in 43.1% of our patients and the incidence of new postoperative permanent deficits was very low, emphasizing the role of brain metastasis resection in oncological treatment. Analogous to gliomas, the EOR of brain metastases has recently become a research focus, although only data from retrospective studies are available to date (10, 13, 30).

### Extent of Resection in Brain Metastases

The incidence of GTR was 80.6% in our cohort, but numbers of so far published retrospective MRI studies varied widely (ranging from 61% to 86%) (10, 11, 13, 30, 31). Different influencing factors are conceivable, such as heterogeneous patient populations, varying use of neuronavigation, neuromonitoring, sodium fluorescein, and intraoperative imaging (iopMRI, iopUS). In addition, the methods used to assess the EOR differed in those studies or were not defined precisely. In the present series, all cases were discussed in an interdisciplinary board and were reviewed by an independent neuroradiologist with many years of experience.

Recently, it was shown that the surgeon’s estimation was misleading in 29-40% of brain metastases cases, thus recommending the routine use of postoperative MRI to assess the EOR (10, 11). Kamp et al. showed in a retrospective analysis of 116 patients an increased risk of local recurrence, if residual tumor was present in the postoperative MRI (OR 8.2; p <.001) (13). Another retrospective study of 64 patients demonstrated a shortened mean survival of 5.6 months in patients with STR compared with 12 months in patients with GTR (p = .025) (10). Another retrospective study of 373 patients reported the same figures, underscoring the importance of GTR in improving survival (30). In contrast, one report found no significant association between STR and shorter survival, indicating the need for prospective studies (32). Additionally, there is currently no study on whether patients with oligo- or multiple metastases benefit from a GTR. In the previous studies, there are also no data on a cut-off postoperative tumor volume/EOR_rel_ indicating re-resection, which may also explain the low re-resection rate in our cohort.

In this study, we identified the following risk factors for uSTR: subcortical metastasis (≥5mm distant from cortex), diffuse contrast agent patterns, contact to the falx/tentorium and non-transcortical approaches. Surgical view can be limited in cases with contact to the falx/tentorium and when using non-transcortical approaches, since tumor tissue can be located behind these structures/”behind the corner”. Thus, the chosen surgical approach also influences the EOR. In patients with cystic/necrotic metastasis, the incidence of uSTR was higher, as it was in a similar retrospective study (11). After opening the tumor cyst/resecting the necrosis, collapse of the tumor walls may occur, which could cause brain shift and increase the risk for uSTR. In contrast, a motor/language associated localization was not associated to a higher risk for uSTR, since we analyzed iSTR separately. Risk stratification and standardized surgical planning with nTMS and DTI tractography are well established in our department for balancing between optimizing the EOR and preventing new neurological deficits (22, 26). The use of these technologies in those challenging cases showed improved functional outcome as well as EOR at the same time (33, 34). Standardized criteria for the use of IOM, particularly in high-risk cases, may explain why it was not substantially associated with EOR in this cohort. Interestingly, patients with a larger tumor volume had no higher risk of uSTR in our series, which contrasts with the results of the aforementioned study (11). The differences may be related to selection bias in their retrospective analysis and to different patient populations – e.g. the median tumor volume and the rate of GTR differed. The multivariate regression tree analysis revealed subcortical metastases (≥5mm distant from cortex) and diffuse contrast agent enhancement to be the most important risk factors. Thus, the probability of uSTR (ranging from 4.8% to 43.2%) can be estimated preoperatively for the first time, thereby determining the necessity for intraoperative imaging to evaluate for residual tumor.

### Intraoperative Imaging

The iopMRI has become an established intraoperative imaging technique for glioma resections showing an improved EOR and progression-free survival (15, 35, 36). To date, resection control of brain metastases by iopMRI has not been investigated. The use of iopMRI is associated with some limitations, since it requires technical, personnel and financial effort, which necessitates a rational, resource-oriented patient allocation (17). Additionally, iopMRI prolongs operating times and causes a large logistical burden, so not all tumor resections can be performed with iopMRI, especially since it is not available in all hospitals (37, 38).

Several observational studies have evaluated iopUS as imaging technique for real-time guidance during brain tumor resection and for improving the EOR (14, 39). The easy and cost-effective application are very important advantages in addition to the ability to localize the tumor despite the intraoperative brain shift (39, 40). However, the use of intraoperative ultrasound is accompanied by the common limitations such as high inter-operator variability, necessary experience, as well as image artifacts occurring in the course of surgery, which can hinder residual tumor detection (39, 41).

Another intraoperative imaging technique is the application of 5-aminolevulinic acid (5-ALA) which is converted to the fluorescent agent protoporphyrin IX and allows selective visualization of tumor tissue after blue-light illumination (42). Similar to iopMRI and iopUS, the use of 5-ALA for resection guidance was mainly investigated in high-grade glioma and showed improved EOR as well as improved (overall) survival (43). Only a few studies examined 5-ALA in brain metastases, but the rate of 5-ALA derived fluorescence was significantly lower (ranging between from 40.5% to 69%) compared to glioma, with no correlation to location or subtype (44–46). Thus, routine use of 5-ALA for resection guidance of brain metastases is severely limited.

Sodium fluorescein is another widely used fluochrome which is extravasated at locations harboring breached blood-brain barrier (e.g. in brain tumors) and equally demonstrated an advantage for EOR as well as survival (47, 48). In contrast to 5-ALA, sodium fluorescein provided higher fluorescence rates in brain metastases (ranging from 90% to 95%), making it more suitable for routine metastases resection guidance (49, 50). A prospective study is warranted to assess whether the incidence of uSTR can be reduced by standard application of sodium fluorescein in high-risk patients.

### Limitations

The current analysis allows for a preoperative MRI-based risk assessment for uSTR. Unfortunately, the use of fluochrome was not part of the prospective study protocol, so we cannot evaluate its benefit for the EOR. Due to the monocenter study design, center-specific decisions and treatments may have influenced the results. No conclusions can be drawn whether local recurrences are more frequent in patients with uSTR and whether this impacts oncological outcome. For this purpose, only retrospective data are available to date, demanding prospective studies to confirm the relevance of intraoperative imaging or re-resections. Moreover, systemic disease burden must be considered in the survival analysis of these patients as local (brain) tumor control is only one variable. Our analysis is limited by the small sample size in some subgroups (e.g., contact with tentorium/falx), therefore some statistically significant changes may have been missed. Future studies may comparatively investigate the role of individual imaging modalities such as iopMRI, iopUS, and sodium fluorescein in reducing the risk for uSTR.

## Conclusions

Based on the preoperative MRI, the risk for uSTR in brain metastases can be estimated in a simple way for clinical routine. Subcortical metastases (>5mm distant from cortex) with diffuse contrast enhancement had the highest risk for an uSTR at 43.2% in contrast to cortical metastases at 4.8%. Thus, the utilization of intraoperative imaging techniques can be decided on an individual basis, whereas the efficiency of these should be investigated in the future.

## Data Availability Statement

The datasets presented in this article are not readily available because of restrictions due to the Data Protection Act. Requests to access the datasets should be directed to TR.

## Ethics Statement

The studies involving human participants were reviewed and approved by ethics committee of the Charité – Universitätsmedizin Berlin; EA2/232/20. Written informed consent for participation was not required for this study in accordance with the national legislation and the institutional requirements.

## Author Contributions

Substantial contributions to the conception or design of the work: TR, JO, MM, PV. Data acquisition: TR, PP, H-CB. Analysis or interpretation of data for the work: TR, UG, JO, PV. Drafting the work or revising it critically for important intellectual content; TR, PP, DW, H-CB, UG, TP, PV. All authors contributed to the article and approved the submitted version.

## Funding

The authors acknowledge the support of the Cluster of Excellence Matters of Activity. Image Space Material funded by the Deutsche Forschungsgemeinschaft (DFG, German Research Foundation) under Germany´s Excellence Strategy – EXC 2025. Dr. Rosenstock is participant in the BIH Charité Digital Clinician Scientist Program funded by the Charité – Universitätsmedizin Berlin, and the Berlin Institute of Health at Charité (BIH).

## Conflict of Interest

The authors declare that the research was conducted in the absence of any commercial or financial relationships that could be construed as a potential conflict of interest.

## Publisher’s Note

All claims expressed in this article are solely those of the authors and do not necessarily represent those of their affiliated organizations, or those of the publisher, the editors and the reviewers. Any product that may be evaluated in this article, or claim that may be made by its manufacturer, is not guaranteed or endorsed by the publisher.
