# Supplementary material for: MRI-Based Risk Assessment for Incomplete Resection of Brain Metastases
Source: Front Oncol. 2022 May 16;12:873175. doi: 10.3389/fonc.2022.873175 (PMC9149256; doi:10.3389/fonc.2022.873175)
Supplement: Supplementary file 1 [file Table_1.docx]

**Supplement 1. Metastases Characteristics of the Intended Subtotal Resections**

|  |  |
| --- | --- |
|  | **total** |
| **n** | **6** |
| **Side of resected metastases**  Right  Left  Midline | 1 (16.7%)  3 (50.0%)  2 (33.3) |
| **Location**  Frontal  Parietal  Cerebellar  Occipital  Temporal  Other | 1 (16.7%)  1 (16.7%)  0  0  1 (16.7%)  3 (50.0%) |
| **Volume**  ≤5ml  ≤10ml  ≤15ml  >15ml | 0  2 (33.3%)  1 (16.7%)  3 (50.0%) |
| **Recurrent metastasis**  No  Yes | 4 (66.7%)  2 (33.3%) |
| **Cystic / necrotic parts**  No  Yes | 3 (50.0%)  3 (50.0%) |
| **Bleeding**  No  Yes | 4 (66.7%)  2 (33.3%) |
| **Edema**  No  Yes | 2 (33.3%)  4 (66.7%) |
| **Distance to ventricle**  ≤5mm  >5mm | 6 (100%)  0 |
| **Contact to falx / tentorium**  No  Falx  Tentorium | 6 (100%)  0  0 |
| **LMD**  No  Yes | 6 (100%)  0 |
| **Occlusive hydrocephalus**  No  Yes | 6 (100%)  0 |
| **Contrast agent patterns**  Diffuse  Circumscribed | 6 (100%)  0 |
| **Cortical vs. subcortical location**  Cortical  Subcortical | 3 (50.0%)  3 (50.0%) |
| **Distance from cortex**  0mm  <5mm distant cortex  <10mm distant cortex  <15mm distant cortex  ≥15mm distant cortex | 3 (50.0%)  0  0  1 (16.7%)  2 (33.3%) |
| **Tumor-cortex angle**  0 - ≤37.5°  37.5% - ≤47°  47° - ≤62.5°  >62.5° | 1 (50.0%)  0  0  1 (50.0%) |
| **Surgical Approach**  Cortical Metastasis  Corticotomy  Interhemispheric  Subfrontal / subtemporal  Other (e.g. retrosigmoidal) | 3 (50.0%)  0  1 (16.7%)  0  2 (33.3%) |
| **Resection guided by Fluorescein sodium**  No  yes | 4 (66.7%)  2 (33.3%) |
| **Motor Associated Location**  No  Yes | 2 (33.3%)  4 (66.7%) |
| **Language Associated Location**  No  Yes | 6 (100%)  0 |

Supplement 1 analyzes the metastases characteristics of the intended subtotal resections. The tumor-cortex angles were only calculated for subcortical metastases whereas no measurement could be performed in one case due to skull base infiltration. **GTR** = gross total resection. **LMD** = leptomeningeal disease.
